# Translational Detection of Indole by Complementary Cell-free Protein Synthesis Assay

**DOI:** 10.3389/fbioe.2022.900162

**Published:** 2022-05-13

**Authors:** You Jin Lee, Soojin Lee, Dong-Myung Kim

**Affiliations:** ^1^ Department of Chemical Engineering and Applied Chemistry, Daejeon, Korea; ^2^ Department of Microbiology and Molecular Biology, Chungnam National University, Daejeon, Korea

**Keywords:** on-site analysis, indole, metabolites, cell-free protein synthesis, personal glucose meter

## Abstract

The information encoded in a single copy of DNA is processed into a plethora of protein molecules via the cascade of transcription and translation. Thus, the molecular process of gene expression can be considered an efficient biological amplifier from the viewpoint of synthetic biology. Cell-free protein synthesis (CFPS) enables the implementation of this amplification module for *in vitro* analysis of important biomolecules and avoids many of the problems associated with whole cell-based approaches. Here, we developed a method to analyze indole by using a combination of enzymatic conversion of indole and amino acid-dependent CFPS. In this method, indole molecules in the assay sample are used to generate tryptophan, which is incorporated into signal-generating proteins in the subsequent cell-free synthesis reaction. The activity of cell-free synthesized proteins was successfully used to estimate the indole concentration in the assay sample. In principle, the developed method could be extended to analyses of other important bioactive compounds.

## Introduction

Most presently used biosensors rely on target-specific binding of purified biomolecules and commonly require complicated fabrication steps to integrate the sensing surface with separate transducers and amplifiers in order to generate readable outputs (Cho et al., 2021; Lim et al., 2021). On the other hand, microbes can sense chemical compounds and generate amplified signals using the protein synthesis machinery and regulatory components. Various microbes can be genetically engineered into stand-alone microbial biosensors that generate biological signals in response to target analytes. However, the widespread application of microbial sensors has been restricted by the intrinsic limitations of using live cells, including the requirement for time-consuming cell culture and conditioning steps. The slow response due to decelerated membrane diffusion of analytes is also a major drawback of microbial sensors (Su et al., 2011; Chen et al., 2020). These limitations of whole cell-based biosensors can be addressed by employing cell-free protein synthesis (CFPS) as an *in vitro* module to express reporter proteins (Lee and Kim, 2019; Voyvodic and Bonnet, 2020; Zhang et al., 2020). Unlike cell-based gene expression methods, CFPS can be directly programmed with the reporter genes without complicated cloning procedures. A CFPS-based biosensor can also be readily modularized to generate reporter proteins in response to target molecules and interfaced with a wide array of analytical devices (Gräwe et al., 2019; Lopreside et al., 2019; Thavarajah et al., 2020; Nguyen et al., 2021). Due to these advantages, the implementation of CFPS as a signal generation module to measure important metabolic compounds has been explored (Liu et al., 2020; McNerney et al., 2020; Silverman et al., 2020). For example, the complementary cell-free protein synthesis (CCFPS) assay was recently developed to detect amino acids and related metabolites (Jang et al., 2017; Jang et al., 2019). In this assay, a reaction mixture for CFPS is prepared in the absence of the amino acid to be analyzed. The lack of an amino acid prevents the reaction mixture from producing full-length proteins, while addition of an assay sample containing the missing amino acid completes the set of 20 amino acids and thereby allows the generation of the reporter protein encoded by the template DNA. The yield of the reporter protein linearly correlates with the titer of amino acids; therefore, the CCFPS assay enables precise measurement of amino acids without complicated chromatographic separation procedures. In addition to being the building blocks of proteins, the synthesis and degradation pathways of amino acids are closely interlinked with the metabolism of diverse compounds. Therefore, these compounds can be enzymatically converted into proteinogenic amino acids for their measurement by the CCFPS assay (Lim et al., 2020).

Indole is an important heterocyclic compound that works as a nucleus for the synthesis of many key compounds in the pharmaceutical (Kaushik et al., 2013; Sravanthi and Manju, 2016; Kumari and Singh, 2019), agricultural (Ye et al., 2021), plastics (Wang et al., 2018; Arza and Zhang, 2019), and perfumery industries (Rodrigues et al., 2021). In addition, indole functions as a major intercellular signaling molecule within the gut microbial ecosystem, and its biosynthesis is an important phenotypic characteristic that can be used to differentiate, identify, and diagnose enteric bacterial infections (Jaglin et al., 2018). Moreover, plasma levels of indole and its derivatives are associated with anxiety-like behaviors in rats, and inflammatory bowel disease and neurological disorders in human (Roager and Licht, 2018). Assays of indole presently involve the performance of gas chromatography or high-performance liquid chromatography (HPLC) in tandem with mass spectrometry, which requires expensive equipment with a large footprint, complicated operational procedures, and trained operators (Dehnhard et al., 1993). Indole is a metabolic precursor of tryptophan; therefore, we envisioned that it can be measured using a CCFPS assay after being converted to tryptophan. To this end, we incorporated an enzymatic reaction that converts indole to tryptophan into the CCFPS assay of tryptophan (Trp-CCFPS assay). The reaction mixture for the Trp-CCFPS assay could produce the reporter protein in response to indole, especially when the cell extract was supplemented with the recombinant *β* subunit of *Pyrococcus furiosus* tryptophan synthase (*Pf*TrpB) (Buller et al., 2015). When the CCFPS assay was programmed with different reporter genes, the activity of the cell-free synthesized proteins exhibited a linear correlation with the indole concentration. In particular, the CCFPS reaction could be programmed with the gene encoding invertase to generate glucose as the signaling molecule in the CCFPS assay. This allowed the indole titer to be read with a personal glucose meter (PGM), which markedly improved the convenience and accessibility of the assay. Taken together, using indole as a model compound, our results demonstrate that the principles of cell-free synthetic biology can be harnessed to build a customized molecular converter that transforms target analytes into readily measurable reporter proteins. The important advantage of this approach is that it can be readily configured to measure the target analytes using various devices available in laboratory settings.

## Materials and Methods

### Materials

Oligonucleotides were synthesized by Macrogen (Seoul, Korea). The gene encoding *Pf*TrpB (Buller et al., 2015) was synthesized by Integrated DNA Technologies (Coralville, IA, United States). DNA polymerase and other reagents for PCR were purchased from Bioline (London, United Kingdom). Restriction enzymes and T4 ligase were obtained from Enzynomics (Daejeon, Korea). Luria-Bertani (LB) medium and ampicillin were purchased from Duchefa Biochemie (Haarlem, Netherlands). Creatine phosphate and creatine kinase were purchased from Roche Diagnostics (Mannheim, Germany). All other chemical reagents and human serum were obtained from Sigma-Aldrich (St. Louis, MO, United States). The S12 extract for CFPS was prepared from the *Escherichia coli* (*E. coli*) strain BL21star (DE3) (Invitrogen, Carlsbad, CA, United States) and diafiltered to remove residual amino acids. To remove residual amino acids, the S12 extract was centrifuged in a Vivaspin centrifugal concentrator (Sartorius Stedim Biotech GmbH, Göttingen, Germany) installed with a 50 kDa molecular weight cutoff membrane. After diluting 2 ml of the extract with 13 ml of the S12 buffer (20 mM Tris−acetate, 28 mM magnesium acetate, and 120 mM potassium acetate, pH 8.2), the diluted S12 extract was concentrated back to its original volume by centrifuging the concentrator at 2,000 × *g*. This process was repeated five times, and the washed S12 extract was stored in a deep freezer in aliquots (Kim et al., 2006; Jang et al., 2019).

### Preparation of Recombinant *Pyrococcus furiosus* Tryptophan Synthase

The chemically synthesized gene encoding *Pf*TrpB was cloned into the pET21a vector to create the plasmid pET21a TrpB. The *E. coli* strain BL21 (DE3) transformed with pET21a TrpB was grown at 37°C in 2.5 L baffled flasks containing 500 ml LB medium. Expression of the recombinant enzyme was induced by adding 0.5 mM isopropyl β-D-1-thiogalactopyranoside (IPTG) when the OD600 of the culture broth reached 0.6. After overnight culture at 30°C following IPTG induction, the cells were harvested by centrifugation (4,500 × *g*, 20 min) and washed thrice with deionized water. After the final wash, cell pellets were resuspended in 20 ml equilibrium buffer (50 mM phosphate buffer, pH 8.0, and 300 mM NaCl) and disrupted by a single passage through a French press (Thermo Fisher Scientific, Waltham, MA, United States) at 12,000 psi. After centrifugation at 12,000 × g for 20 min, recombinant *Pf*TrpB in the supernatant was purified using a Ni-NTA agarose column.

### Complementary Cell-Free Protein Synthesis Assay to Measure Indole

The reaction mixture for the indole assay consisted of the following components in a final volume of 30 μL: 57 mM HEPES-KOH (pH 8.2), 1.2 mM ATP, 0.85 mM each of CTP, GTP, and UTP, 2 mM DTT, 0.17 mg/mL *E. coli* total tRNA mixture (from the strain MRE600), 0.64 mM cAMP, 90 mM potassium glutamate, 80 mM ammonium acetate, 12 mM magnesium acetate, 34 μg/mL L-5-formyl-5,6,7,8-tetrahydrofolic acid, 2 mM each of 19 amino acids excluding tryptophan, 2% polyethylene glycol 8000, 67 mM creatine phosphate, 3.2 μg/ml creatine kinase, 27% (v/v) diafiltered S12 extract, and 6.7 μg/ml template DNA encoding *E. coli* invertase between the T7 promoter and T7 terminator (Dehnhard et al., 1993; Jang et al., 2017). In experiments using purified *Pf*TrpB, the assay mixture was supplemented with 100 mM Tris-HCl (pH 8.0), 180 mM NaCl, and 10 μM pyridoxal phosphate (PLP) (Cellini et al., 2020). After addition of indole at various concentrations, the assay mixture was incubated at 30°C for 90 min. To analyze superfolder green fluorescent protein (sfGFP) produced during the CCFPS assay, 15 μL of the completed assay reaction was diluted with 200 μL phosphate-buffered saline (PBS) and fluorescence was measured with a Victor X2 microplate reader (PerkinElmer, Waltham, MA, United States). In the CCFPS assay programmed with the invertase gene, 15 μL of the completed assay mixture was transferred to an Eppendorf tube containing 15 μL of 200 mM sucrose prepared in PBS and further incubated for 10 min at 37°C. After heat-inactivation of invertase, the glucose titer was measured in 5 μL of the supernatant from the briefly centrifuged reaction mixture using a PGM (Accu-Check Inform II, Roche Diagnostics). Limit of detection (LOD) for each experiment was calculated based on the standard deviation of the response (Sy) of the curve and the slope of the calibration curve (S) at levels approximating the LOD according to the formula: LOD = 3.3(Sy/S).

## Results and Discussion

### Conversion of Indole to Tryptophan in *E*. *coli* Extract

**FIGURE 1:**
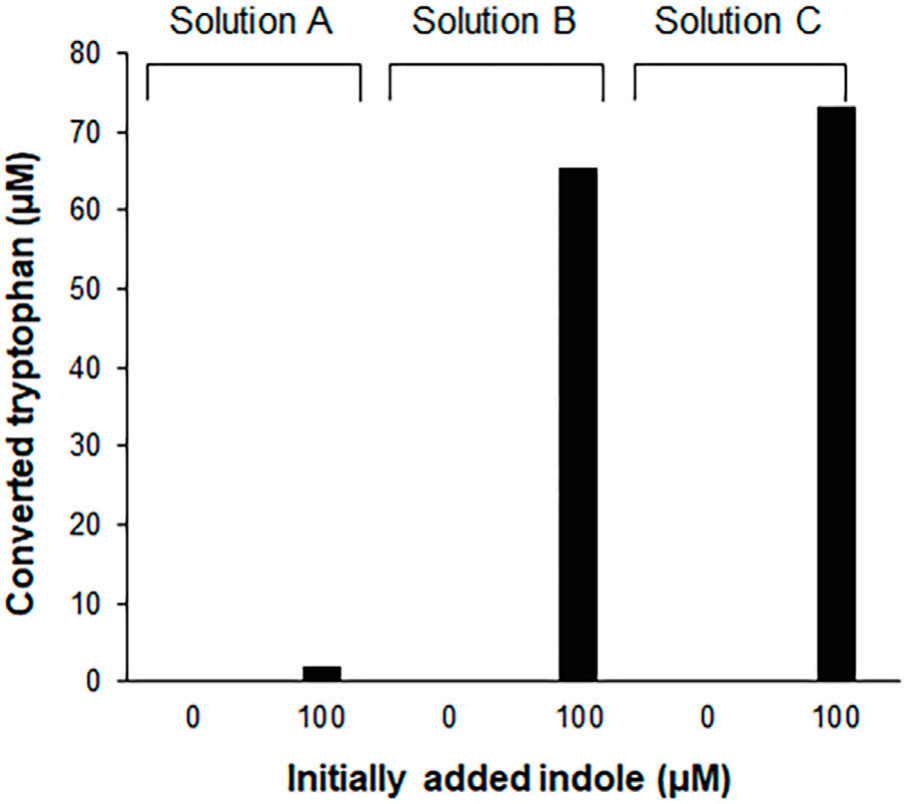
Conversion of indole to tryptophan in the S12 extract solutions. 100 μM indole was incubated for 2 h at 30°C, in the S12 extract diluted in different solutions [27% (v/v)]. Solution A, S12 extract diluted in PBS; solution B, S12 extract diluted in the reaction mixture containing CFPS components prepared in the absence of tryptophan and template DNA; solution C, solution B without amino acids. Concentrations of tryptophan converted from indole were measured using a Hitachi L-8900 amino acid analyzer (Hitachi High-Technologies, Tokyo, Japan) following the manufacturer’s protocols.

Tryptophan contains an indole ring attached to the alanyl side chain. As shown in Supplementary Figure S1, biosynthesis of tryptophan from chorismate involves five enzymes. Among these enzymes, tryptophan synthase, a tetrameric enzyme consisting of αββα subunits, catalyzes the final two steps of tryptophan biosynthesis. The *α* subunit (TrpA) catalyzes the cleavage of indole-3-glycerol phosphate to form indole and glyceraldehyde-3-phosphate, while the *β* subunit is responsible for the condensation of indole with serine to produce tryptophan. This is an endogenous pathway in *E. coli*; therefore, we tested if the S12 extract used for the CCFPS assay can convert indole to tryptophan. As expected, HPLC analysis revealed that incubation of indole in the S12 solution led to accumulation of tryptophan (Figure 1). However, the conversion yield of indole to tryptophan varied dramatically depending on the solution in which the S12 extract was diluted. For example, the conversion yield in the S12 extract diluted in PBS (solution A in Figure 1) did not exceed 5% after incubation for 2 h. By contrast, the same amount of S12 extract converted more than 60% of indole to tryptophan when it was diluted in reaction mixture containing CFPS components prepared in the absence of tryptophan and template DNA (solution B in Figure 1). Tryptophan is a condensation product of indole and serine; therefore, we first reasoned that the lower conversion yield of indole by the PBS-diluted S12 extract was due to the lack of serine, which was abundantly supplied in the CFPS mixture. However, contrary to this expectation, removal of amino acids from solution B did not lower the conversion yield of indole (solution C in Figure 1). This indicates that there was a substantial amount of residual serine in the S12 extract and that conversion of indole by the S12 extract was affected by a different component of solution B. Although it was not further investigated which component(s) in solution B affected the conversion of indole to tryptophan, this result indicates that the assay mixture of CFPS provides an environment that substantially enhances the conversion of indole to tryptophan.

### Measurement of the Indole Concentration by the Trp-Complementary Cell-Free Protein Synthesis Assay

**FIGURE 2:**
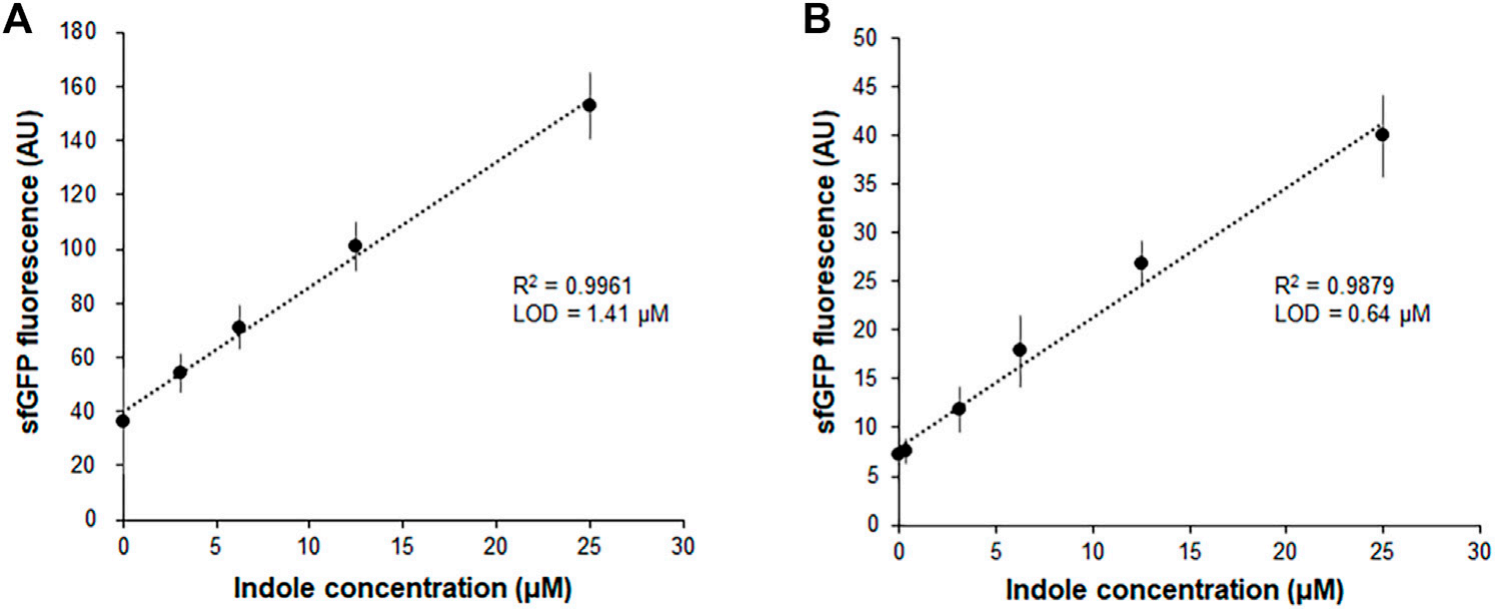
Measurement of indole by the fluorescence of sfGFP synthesized from Indole-CCFPS assay. Indole-CCPFS assay was performed with varying concentrations of indole using standard S12 extract **(A)** or diafiltered S12 extract **(B)**. The fluorescence of the synthesized sfGFP was measured after 2 h incubation at 30°C. Measurements were performed in triplicate, and the error bars represent the standard deviations of three independent experiments.

Our previous report demonstrated that the CCFPS assay is a powerful alternative to existing chromatographic methods for amino acid analysis (Jang et al., 2017; Jang et al., 2019). For instance, without any chemical derivatization and chromatographic separation steps, the Trp-CCFPS assay could detect micromolar concentrations of tryptophan within 90 min (Supplementary Figure S2). HPLC analysis of the aforementioned experiments confirmed that indole was converted to tryptophan by the S12 extract under the conditions used for the CCFPS assay; therefore, we attempted to link the extract-catalyzed conversion of indole with the Trp-CCFPS assay (Indole-CCFPS assay). The initially designed assay mixture for the Indole-CCFPS assay consisted of the same components as were used for the Trp-CCFPS assay. When the Indole-CCFPS mixture programmed with DNA encoding sfGFP was supplied with 100 μM indole, incubation of the assay mixture led to generation of sfGFP fluorescence, as expected (Supplementary Figure S3A). However, the fluorescence intensity was already high without addition of indole. In principle, signal generation during a CCFPS assay should be dependent on exogenous addition of the amino acid missing from the assay solution. However, in practice, the S12 extract contains substantial amounts of amino acids from the cytoplasm of *E. coli*. During the Indole-CCFPS assay, residual tryptophan would cause background synthesis of proteins. The high level of background protein synthesis might affect the sensitivity of the CCFPS assay; therefore, the S12 extract was diafiltered to remove residual tryptophan prior to its use in this assay. Diafiltration of the cell extract markedly lowered the background synthesis of sfGFP during the Indole-CCFPS assay (Supplementary Figure S3B). Although fluorescence observed upon addition of indole was also lowered, due to the markedly reduced background, the signal-to-noise ratio upon addition of 100 μM indole was improved from 4 to 7 (Supplementary Figure S3B). When the Indole-CCFPS assay was performed using the diafiltered S12 extract, the intensity of sfGFP fluorescence exhibited a strong correlation with the indole concentration. Compared with the same assay performed with the standard S12 extract, the use of diafiltered S12 extract substantially lowered the limit of detection for indole (Figures 2A,B).

**FIGURE 3:**
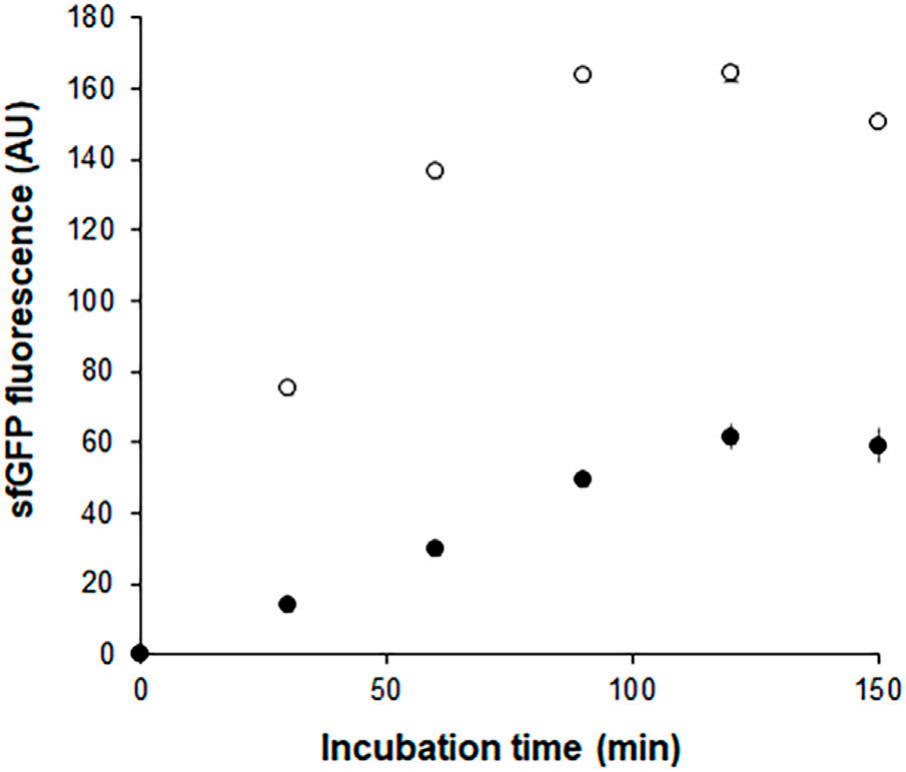
Time courses of sfGFP synthesis during Indole-CCFPS and Trp-CCFPS assays. The CCFPS assay mixtures programmed with the template DNA encoding sfGFP were incubated at 30°C. 15 μL assay mixture was withdrawn at the indicated time points and measured for sfGFP fluorescence after being diluted in 200 μL PBS. Measurements were performed in triplicate, and the error bars represent the standard deviations of three independent experiments.

**FIGURE 4:**
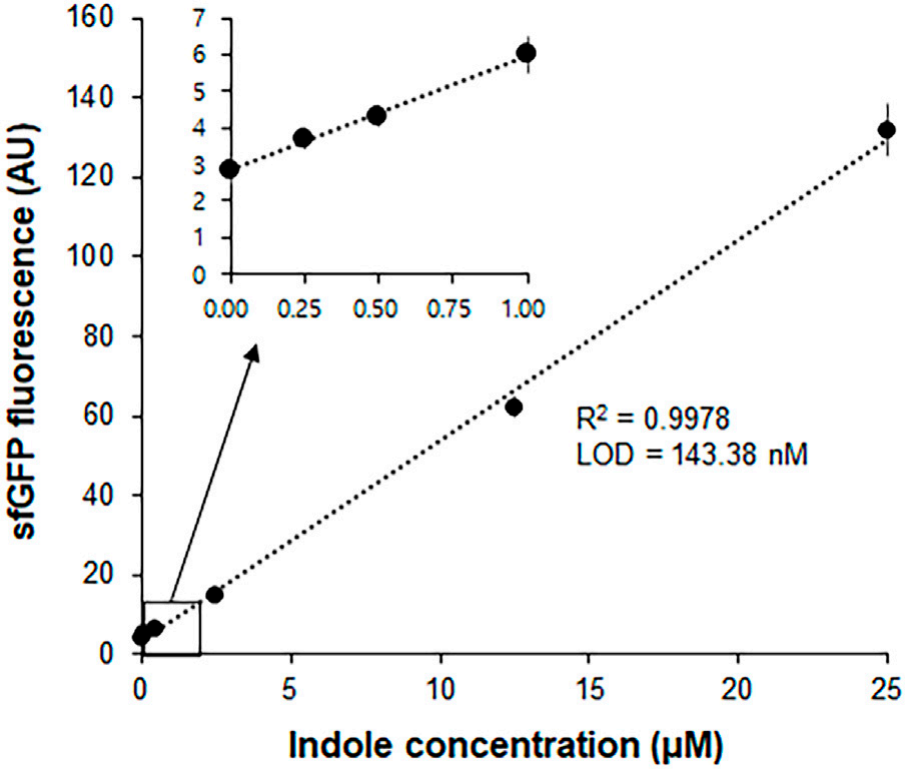
Measurement of indole by Indole-CCPFS assay employing *Pf*TrpB-enriched S12 extract. The S12 extract was prepared after overexpression of the *Pf*TrpB enzyme during the cultivation of *E. coli* cells. The resulting *Pf*TrpB-enriched S12 extract was diafiltered to remove residual amino acids prior to its use for Indole-CCFPS assay programmed with the template DNA encoding sfGFP. Indole-CCFPS assay was performed with varying concentrations of indole, and measured for the fluorescence of synthesized sfGFP. Inset represents the enlarged graph of the boxed region. Measurements were performed in triplicate, and the error bars represent the standard deviations of three independent experiments.

In a separate experiment, we compared the time-courses of sfGFP generation between the Trp-CCFPS and Indole-CCFPS assays. When the same concentrations (100 μM) of tryptophan and indole were tested, both the increase rate and maximum value of sfGFP fluorescence were markedly lower during the Indole-CCFPS assay than during the Trp-CCFPS assay (Figure 3). We reasoned that the rate of indole conversion limited the supply of tryptophan during the Indole-CCFPS assay, resulting in sluggish synthesis of sfGFP. Based on this assumption, to further increase the speed and sensitivity of indole detection, we attempted to accelerate the conversion of indole using recombinant tryptophan synthase. Only the activity of the *β* subunit, not the entire αββα complex of tryptophan synthase, is required for coupling of indole and serine; therefore, the assay mixture for the Indole-CCFPS assay was supplemented with *Pf*TrpB, an engineered *β* subunit of tryptophan synthase of *Pyrococcus furiosus* (Buller et al., 2015). While the activity of natural TrpB decreases when it is separated from the native complex, this variant enzyme carries activating mutations that restore the activity of stand-alone TrpB. As expected, the fluorescence signal from the Indole-CCFPS assay increased in proportion to the amount of supplemented *Pf*TrpB and peaked when 1.0 mg/ml of the recombinant enzyme was used (Supplementary Figure S4). Use of *Pf*TrpB also markedly reduced the amount of time required to perform the Indole-CCFPS assay because sfGFP fluorescence plateaued within 90 min. Separate preparation of purified *Pf*TrpB could be avoided by preparing the S12 extract after overexpressing the enzyme during cultivation of transformed *E. coli* cells. The *Pf*TrpB-enriched extract resulted in a similar rate and yield of indole conversion as the standard S12 extract supplemented with purified enzymes and thus was used in subsequent experiments (Supplementary Figures S4 and S5). With these modifications, the sensitivity of the Indole-CCFPS assay was further improved to measure nanomolar concentrations of indole (Figure 4).

### Measurement of Indole by a Personal Glucose Meter

**FIGURE 5:**
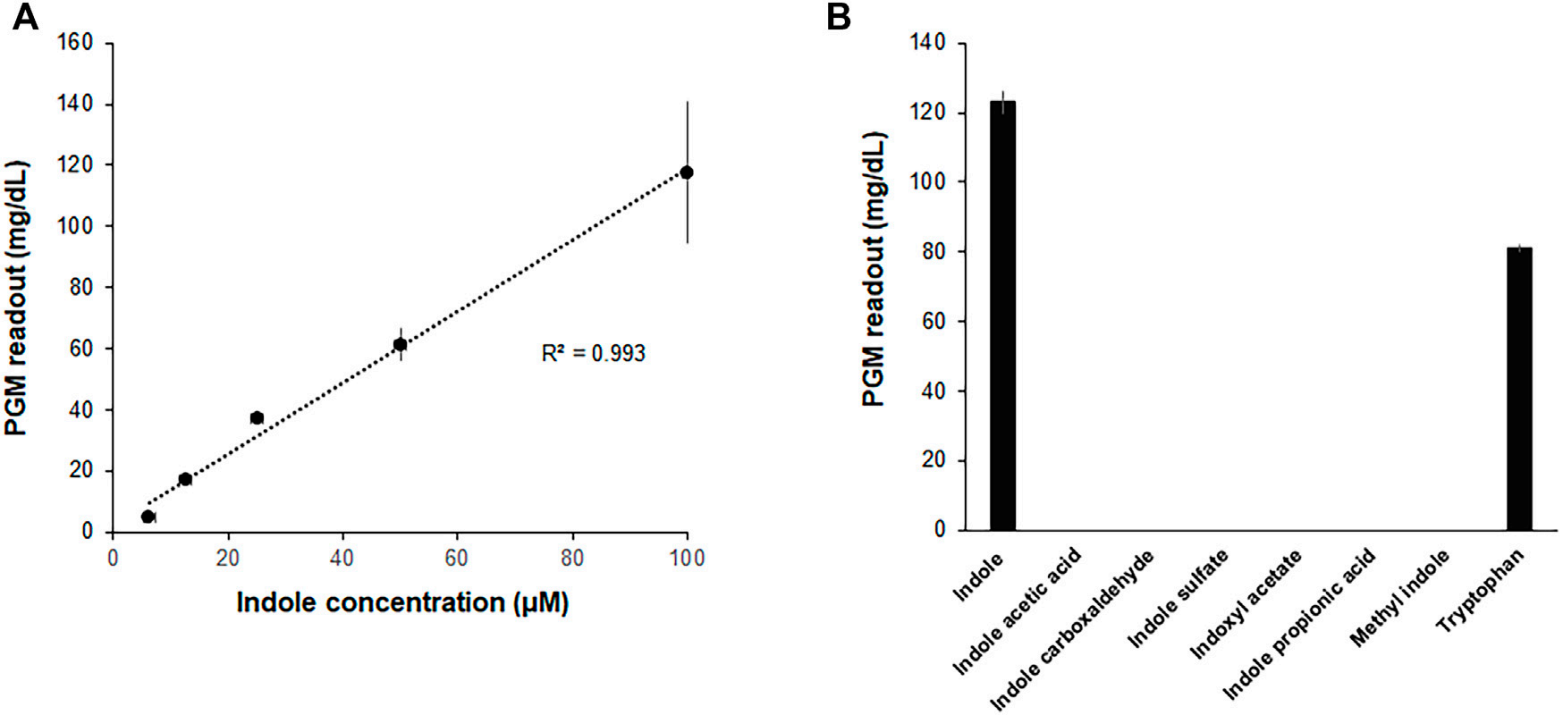
Measurement of indole by a PGM. The assay mixture for Indole-CCFPS was programmed with the template DNA encoding bacterial invertase. 15 μL of the assay mixture was transferred to an Eppendorf tube containing 15 μL of 200 mM sucrose prepared in PBS and further incubated for 10 min at 37°C. After heat-inactivation of invertase, the glucose titer was measured in 5 μL of the supernatant from the briefly centrifuged reaction mixture using a PGM (Accu-Check Inform II, Roche Diagnostics). **(A)** The PGM readouts were measured for varying concentrations of indole. **(B)** The assay mixture for Indole-CCFPS was supplied with the same concentrations (100 μM) of indole, and measured for the glucose concentration using a PGM. Measurements were performed in triplicate, and the error bars represent the standard deviations of three independent experiments.

In an attempt to further improve the convenience and portability of the indole assay, the CCFPS assay was interfaced with glucose measurement by a PGM, the most widely distributed analytical device. To this end, the template DNA for the Indole-CCFPS assay was switched from pK7sfGFP to pK7-inv, which encodes *E. coli* invertase. In addition, 200 mM sucrose was included in the assay mixture to generate glucose in the presence of indole. When the glucose titer in the assay mixture was measured after incubation for 90 min, the readout of the PGM showed almost a linear correlation with the concentration of indole (Figure 5A), indicating that a PGM can be used as a portable device to measure indole concentrations. The Indole-CCFPS assay could also specifically discriminate indole from its derivatives. When seven compounds structurally related to indole were tested in parallel, none of the analogs could generate signals detectable with the PGM in the CCFPS assay (Figure 5B).

### Discriminative Measurement of Tryptophan and Indole in a Mixed Solution

**FIGURE 6:**
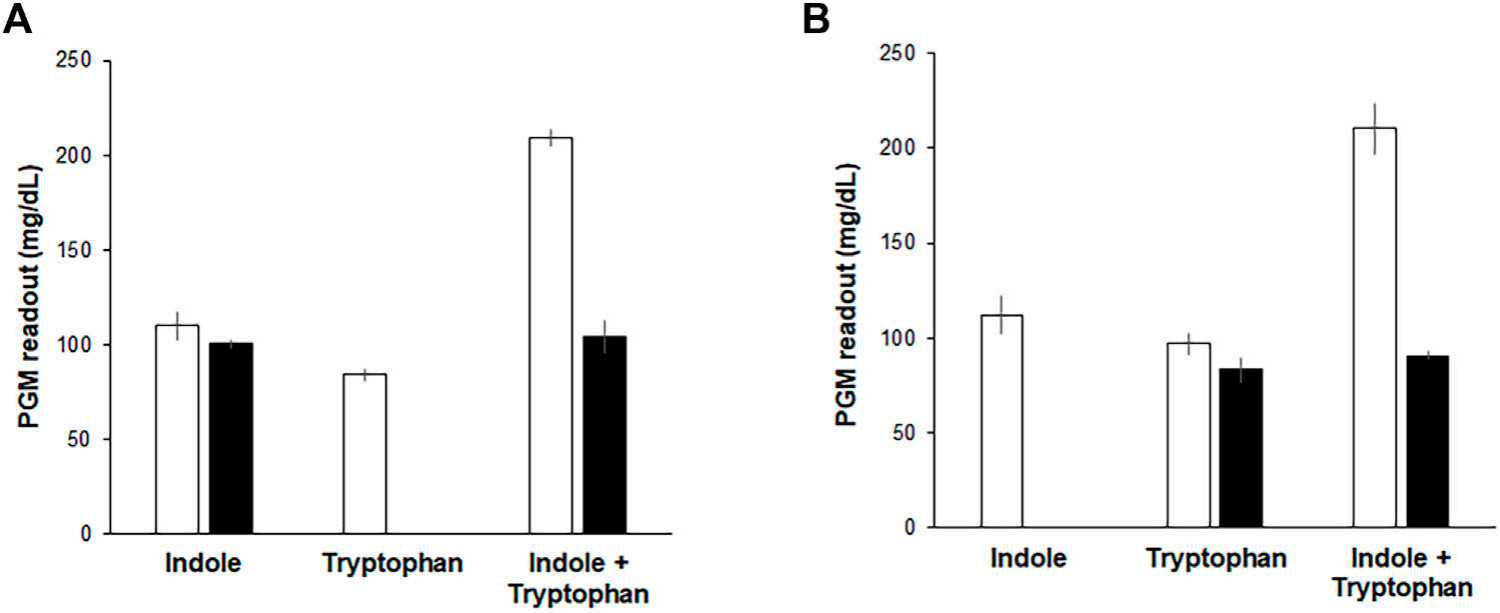
Discriminative measurement of indole and tryptophan. Indole, tryptophan, and their mixture were analyzed by Indole-CCFPS assay using a PGM as a measuring device. **(A)** for the selective detection of tryptophan, the mixed solution of tryptophan and indole was treated with LAAO (filled bars), as described in Materials and Methods. Blank bars represent the results of Indole-CCFPS assays without the LAAO treatment. **(B)** for the selective detection of indole, the assay mixture was supplemented with AOA (filled bars), as described in Materials and Methods. Blank bars represent the results of Indole-CCFPS assays in the absence of AOA. Measurements were performed in triplicate, and the error bars represent the standard deviations of three independent experiments.

Although the above results indicate that the Indole-CCFPS assay can conveniently detect indole with high specificity and sensitivity, it cannot discriminate indole in a mixture with tryptophan, which may be required when analyzing indole in biological samples. This problem was addressed by pre-treating assay samples with L-amino acid oxidase (LAAO). LAAO oxidizes tryptophan to indole-3 pyruvate and thus prevents the incorporation of tryptophan in assay samples into the reporter protein during the Indole-CCFPS assay (Supplementary Figure S6). Incubation with LAAO from *Crotalus adamanteus* almost completely repressed incorporation of tryptophan into sfGFP, but did not affect signal generation by indole (Figure 6A). On the other hand, tryptophan can also be differentiated from indole by repressing the conversion of indole to tryptophan. Aminooxyacetate (AOA) is a potent inhibitor of PLP-dependent enzymes. TrpB is a PLP-dependent enzyme (Cellini et al., 2020); therefore, we expected that conversion of indole to tryptophan would be repressed by including AOA in the CCFPS assay mixture (Supplementary Figure S6). Indeed, when tryptophan and/or indole were added to the assay mixture containing AOA, only tryptophan could be used to synthesize sfGFP (Figure 6B). Taken together, these results demonstrate that tryptophan and indole can be discriminately measured by dividing the mixed solution into two and treating one sample with LAAO and the other with AOA.

## Conclusion

By harnessing the translational machinery of cells as an *in vitro* signal generation module, we developed a method that enables rapid detection of nanomolar concentrations of indole. A few colorimetric methods have been developed to detect indole without using sophisticated instruments. For example, Kovác’s assay uses para-dimethylaminobenzaldehyde, which reacts with indole to generate a red product (Turner, 1961). Darkho et al. also reported a colorimetric method that uses hydroxylamine and can discriminate indole from structurally related compounds (Darkoh et al., 2015). However, these methods can only detect millimolar concentrations and have limited selectivity for indole. In particular, the sensitivity of indole assay needs to be improved to differentiate subtle changes caused by the infection of pathogenic bacteria (Chappell et al., 2016). The amplification nature of protein synthesis employed in the presented method enables the generation of biologically converted and amplified signals, thereby markedly improving the sensitivity of detection. Furthermore, the modular nature of this approach provides great flexibility in terms of how the signal outputs generated from indole are read. For example, by programming the assay with a glucose-generating enzyme, the titer of indole can be measured with a PGM. PGMs are the most widely distributed analytical device around the world and are relatively cheap and easy to use. A method that uses a PGM to analyze analytes of interest will greatly enhance simplicity and convenience, especially in low resource settings with limited laboratory services or where it is hard to access relevant facilities.

## Data Availability

The original contributions presented in the study are included in the article/ **Supplementary Material** , further inquiries can be directed to the corresponding author.

## Fundings

This work was supported by the National Research Foundation (NRF) (2020R1A2C2013114 and 2020R1A5A8017671).
