# Supplementary material for: Translational Detection of Indole by Complementary Cell-free Protein Synthesis Assay
Source: Front Bioeng Biotechnol. 2022 May 13;10:900162. doi: 10.3389/fbioe.2022.900162 (PMC9136167; doi:10.3389/fbioe.2022.900162)
Supplement: Supplementary file 1 [file DataSheet1.docx]

**Supplementary Information**

Translational detection of indole by complementary cell-free protein synthesis assay

You Jin Lee^1^, Soojin Lee^2^, Dong-Myung Kim^1,*^

^1^Department of Chemical Engineering and Applied Chemistry, ^2^Department of Microbiology and Molecular Biology, Chungnam National University, Daejeon, Korea

*** Correspondence:**Dong-Myung Kim
dmkim@cnu.ac.kr

Keywords: on-site analysis, indole, metabolites, cell-free protein synthesis, personal glucose meter.


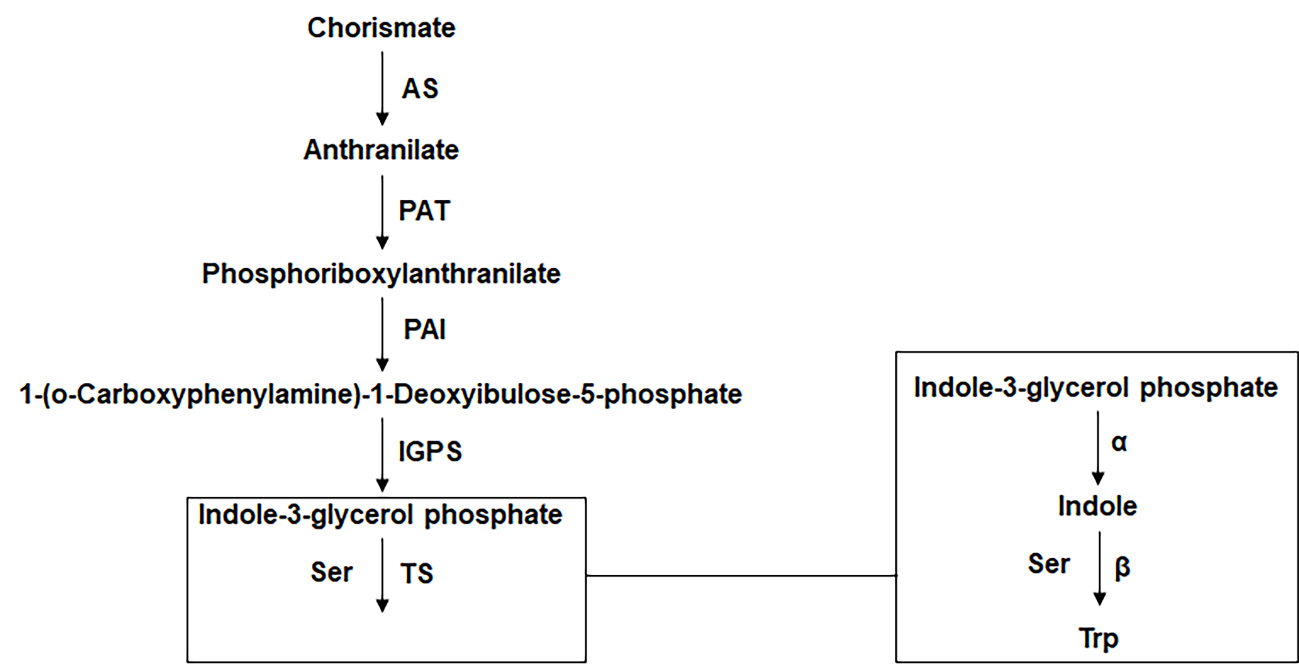


**Figure S1. Biosynthetic pathway of indole.** AS, anthranilate synthase; PAT, phosphoribosylanthranilate transferase; PAI, phosphoribosyl anthranilate isomerase; IGPS, indole-3-glycerol phosphate synthase; Trp, tryptophan; TS, tryptophan synthase; Ser, serine.

**
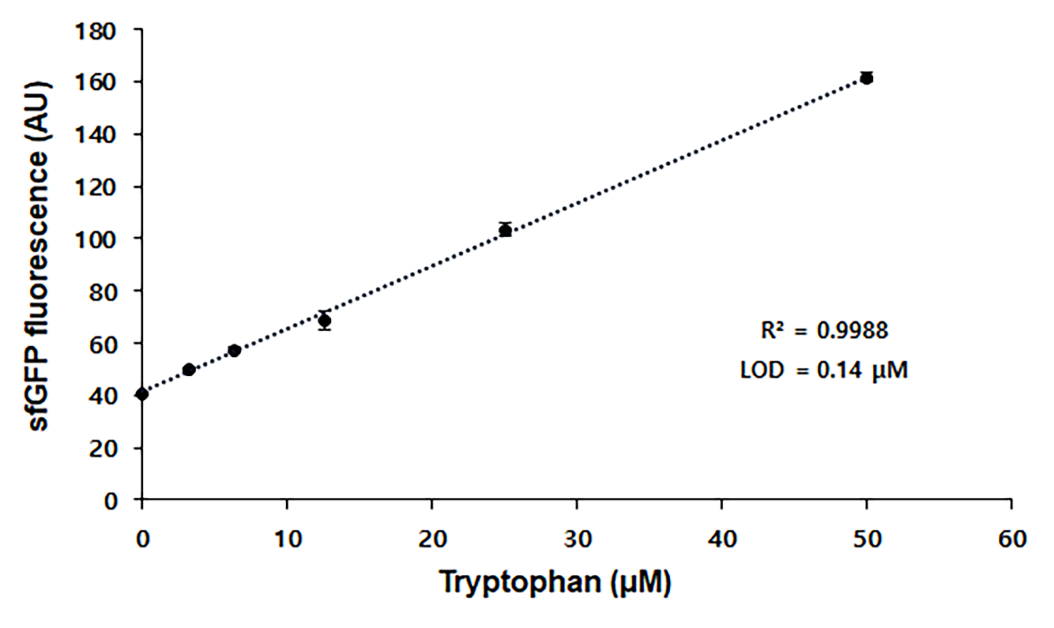
**

**Figure S2. Measurement of tryptophan by Trp-CCFPS assay.** Measurements were performed in triplicate, and the error bars represent the standard deviations of three independent experiments.

**
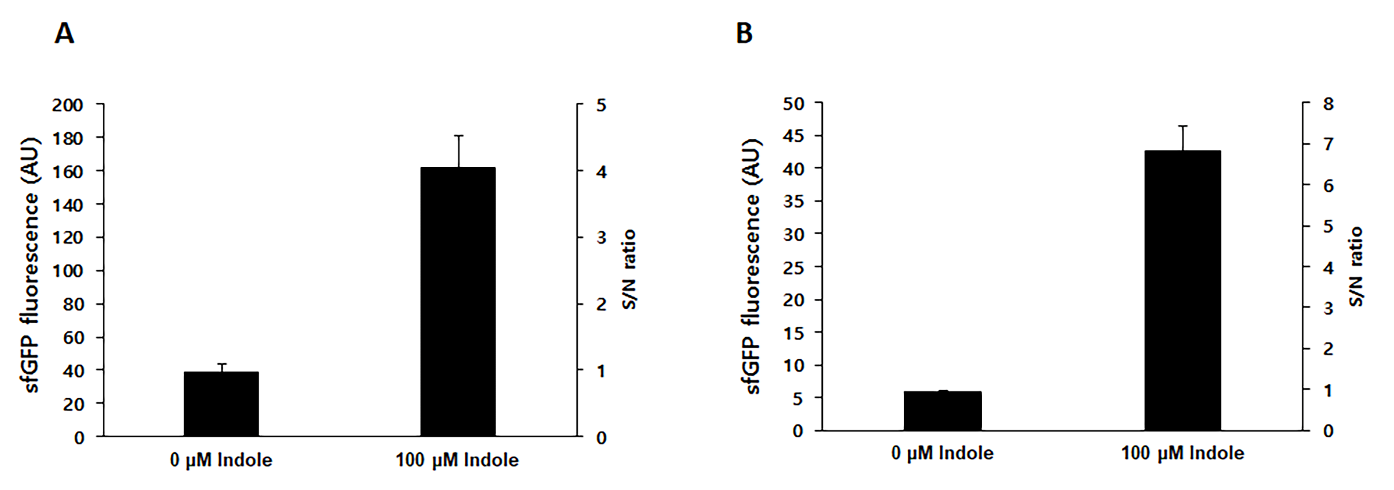
**

**Figure S3. Measurement of indole by Indole-CCFPS assay using diafiltered S12 extract.** The assay mixture for Indole-CCFPS was prepared using the standard (A) and diafiltered (B) S12 extract. The assay mixture was incubated in the presence of 100 μM indole, and measured for the synthesized sfGFP. Although the use of diafiltered S12 extract lowered intensity of sfGFP fluorescence, it markedly reduced the background signal, and thus improved the S/N ratio of the assay. Measurements were performed in triplicate, and the error bars represent the standard deviations of three independent experiments.

**
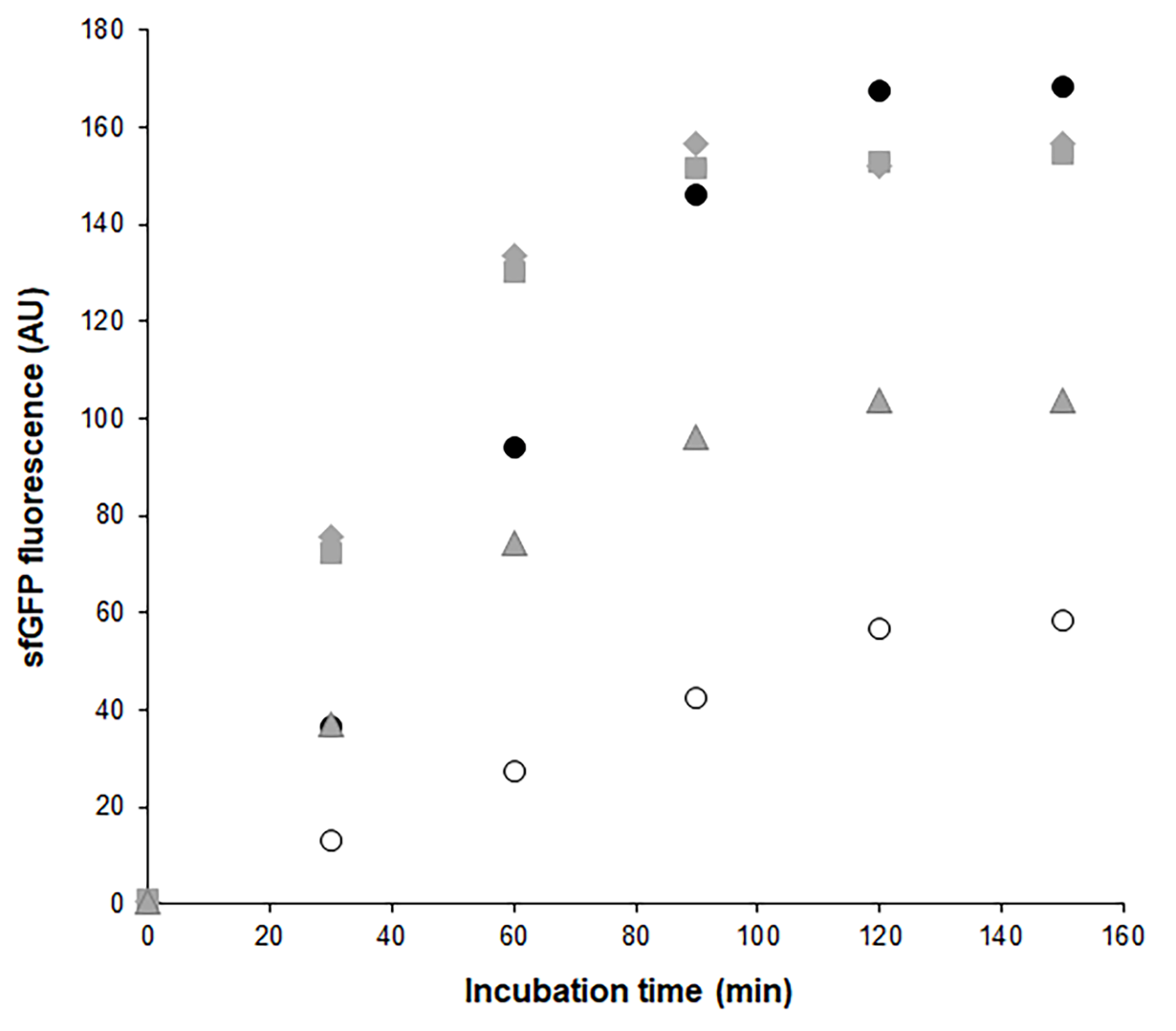
**

**Figure S4. Enhanced synthesis of sfGFP by the supplementation of *Pf*TrpB during Indole-CCFPS assay.** The mixture for Indole-CCFPS was incubated in the presence of 100 μM indole, and the increase of sfGFP fluorescence was monitored over the incubation time. Open circles, Indole-CCFPS assay using diafiltered standard S12 extract; triangles, Indole-CCFPS assay using diafiltered standard S12 extract after supplementation with 0.5 mg/mL of purified *Pf*TrpB; squares, Indole-CCFPS assay using diafiltered standard S12 extract after supplementation with 1.0 mg/mL of purified *Pf*TrpB; diamonds, Indole-CCFPS assay using diafiltered standard S12 extract after supplementation with 2.0 mg/mL of purified *Pf*TrpB; filled circles, Indole-CCFPS assay using diafiltered *Pf*TrpB-enriched S12 extract.


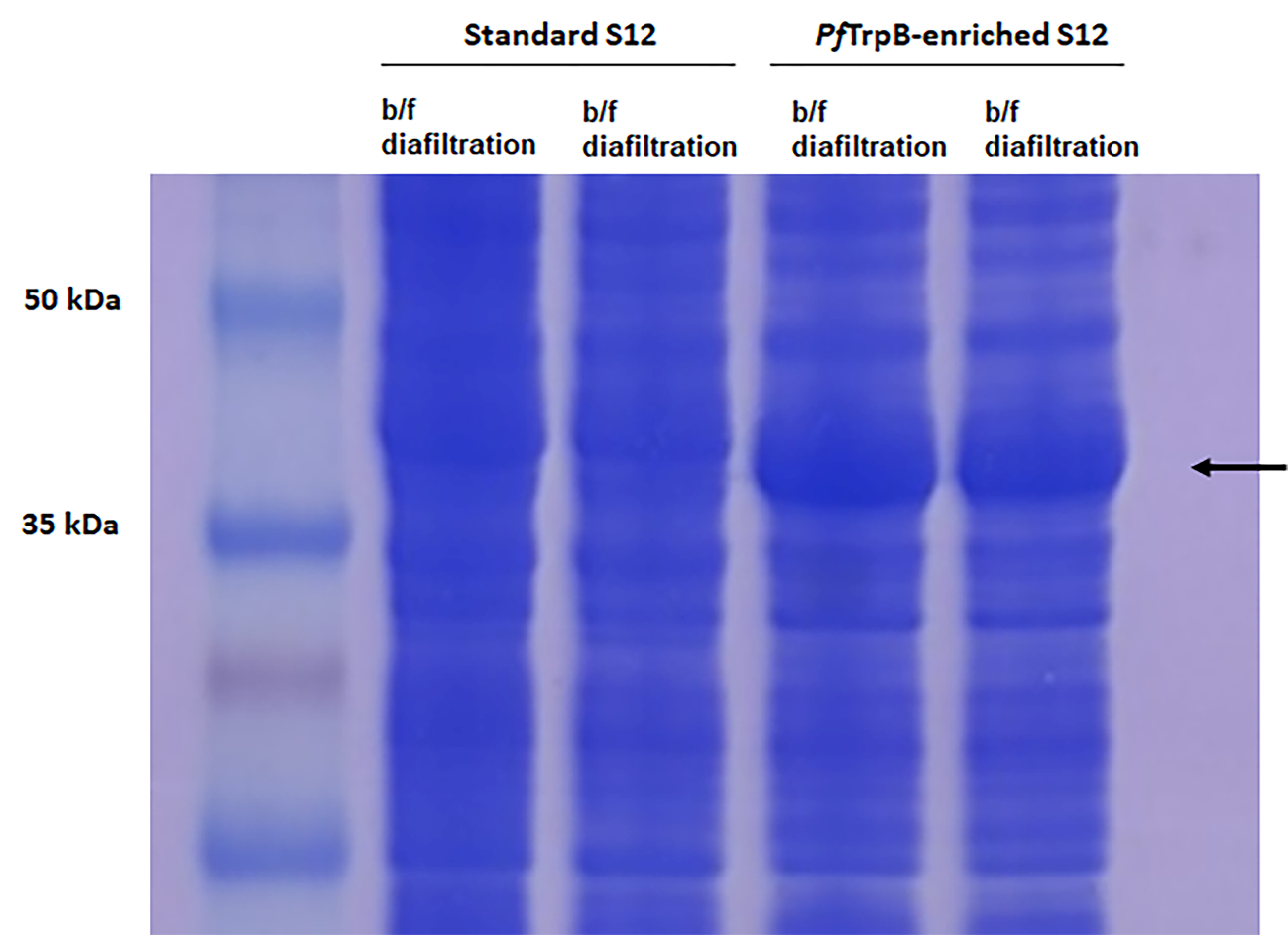


**Figure S5. Preparation of the S12 extract after overexpression of *Pf*TrpB.** The S12 extract was prepared from the *E. coli* strain BL21star (DE3) transformed with the plasmid pET21a TrpB. SDS-PAGE analysis of the resulting S12 extract (*Pf*TrpB-enriched S12) confirmed the overexpression of *Pf*TrpB. Arrow indicates the position of *Pf*TrpB.


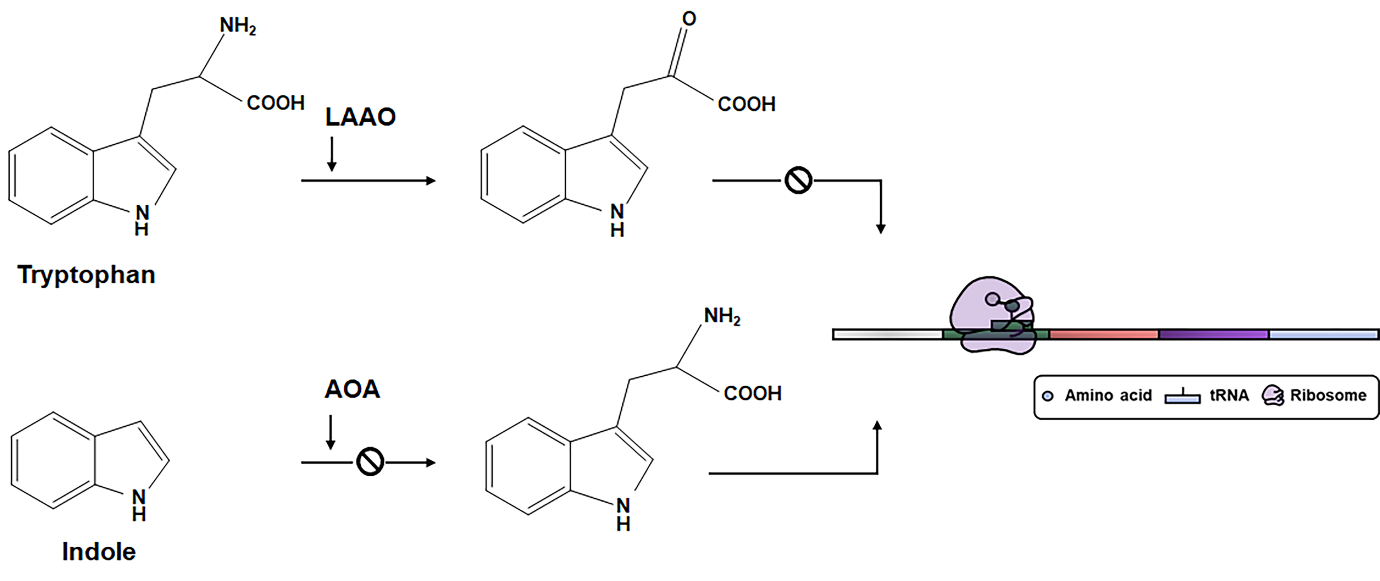


**Figure S6. Scheme for discriminative detection of tryptophan and indole by CCFPS assay.** For the selective detection of indole, the mixed solution of tryptophan and indole were treated with LAAO to oxidize tryptophan. The mixed solution was then analyzed after heat-inactivation of LAAO. For the selective detection of tryptophan, Indole-CCFPS assay mixture was supplemented with AOA, which prevents the conversion of indole to tryptophan.
